# Incidence of chronic osteomyelitis between 2016 and 2022 in a large, multicenter database in the United States

**DOI:** 10.5194/jbji-10-377-2025

**Published:** 2025-10-15

**Authors:** Rawabi Aljadani, Hyunkeun Cho, Martha L. Carvour

**Affiliations:** 1 Department of Epidemiology, University of Iowa College of Public Health, Iowa City, IA 52242, USA; 2 Department of Internal Medicine, Carver College of Medicine, the University of Iowa, Iowa City, IA 52242, USA; 3 Department of Biostatistics, University of Iowa College of Public Health, Iowa City, IA 52242, USA

## Abstract

**Introduction**: Chronic osteomyelitis (COM) is a serious musculoskeletal infection that affects a patient's quality of life and long-term survival. In this study, we assessed overall, regional, and patient-level characteristics of bacterial COM in a large, multicenter database in the United States. **Methods**: We used ICD-10 codes to identify incident bacterial COM in the TriNetX database between 1 January 2016 and 31 December 2022. We calculated COM incidence per 1000 adult patients with the associated 95 % confidence intervals. We used the Cochran–Armitage test to assess incidence trends across the study period. **Results**: A total of 93 324 adult patients were identified. Overall, a steady COM incidence was observed over time, with some indication of lower rates starting in 2020. The incidence was about 2-fold higher in males than females. As expected, lower-extremity COM was most common overall and among males. Although lower-extremity COM and vertebral COM had comparable incidence among females, vertebral COM became slightly more common than lower-extremity COM among females during the study period. **Conclusions**: To our knowledge, this study provides the largest available, multicenter estimate of COM incidence in the United States. Although the incidence of COM was generally steady over time, a slight reduction was noted during the pandemic (2020 and later). This finding may reflect important differences in ascertainment or competing risks during that period.

## 1 Introduction

Chronic osteomyelitis (COM) is a serious infection that impacts a patient's functional status, quality of life, and long-term survival (Akiyama et al., 2013; Lang et al., 2023). COM diagnoses often rely on a combination of clinical, radiological, microbiological, and histopathological features; this diagnostic evaluation and subsequent treatment for COM can be complicated and costly. In the United States, a 12-month average cost for osteomyelitis (OM) with a segmental bone defect has been estimated at USD 156 818 (95 % CI: USD 112 970–217 685) (Norris et al., 2021). This estimate was based on index surgery costs, perioperative costs (including costs associated with bleeding; infectious complications, such as pneumonia; cardiovascular and renal complications; other complications; length of hospital stay; operating room time; and total index hospital costs), and post-operative costs during a 12-month window after surgery (Norris et al., 2021), using the Premier Healthcare and MarketScan databases.

Despite the substantial impacts of COM on patient outcomes and entire health systems, the overall incidence of COM in the United States is not well defined, with many prior studies emphasizing acute or general forms of OM. Previously, population-based OM incidence was reported among residents in Olmsted County, Minnesota, between 1969 and 2009 (Kremers et al., 2015). The study found an overall age- and sex-adjusted OM incidence rate of 21.8 per 100 000 person-years (Kremers et al., 2015). Even though this was the first study to provide an insight into OM incidence at a population level, the results provide limited context about (1) the impact of diagnostic quality over time (extending back to 1969) on OM incidence; (2) the distinction, if any, between acute and chronic OM; and (3) the relationship of competing or contributing risks – such as diabetes and fractures – over time in this population.

Due to the lack of an established OM registry or another comprehensive data source to assess national OM incidence, other studies have emphasized OM-related national hospital admission rates for adults and children, usually for specific anatomic sites (Henke et al., 2005; Shaikh et al., 2021; Schmidt et al., 2023; Issa et al., 2018). Vertebral-OM-related national hospital admissions were estimated at 5.4 admissions per 100 000 patients in 2013 (Henke et al., 2005), while foot- and toe-OM-related hospital admissions were estimated at 9 admissions per 10 000 patients per year (Issa et al., 2018). However, because these estimates identified OM in inpatient settings, the results may underestimate OM diagnosed or treated exclusively in outpatient settings and may, thus, underestimate OM incidence overall.

There is a need to better characterize the epidemiology of COM across centers over time. In this study, we computed bacterial COM incidence rates in a large, multicenter dataset for each year between 1 January 2016 and 31 December 2022 and identified patterns in COM incidence stratified by geographic and patient-level characteristics.

## 2 Methods

### 2.1 Data source and study population

We used the TriNetX network database, a real-world data source based in the United States, which consists of anonymized electronic medical record data from a network of approximately 80 healthcare organizations (TriNetX, 2024). The majority of the network's healthcare organizations are adult acute-care hospitals with many locations and large academic medical institutions with both inpatient and outpatient services (TriNetX, 2024). The study population included all adult patients ( ≥ 18 years) recorded in the multicenter TriNetX network research database between 1 January 2016 and 31 December 2022.

### 2.2 COM case definition, identification, and classification

A bacterial COM case was defined as any of the following ICD-10 codes recorded between 1 January 2016 and 31 December 2022: M86.3-86.6 and M46.2 (Table S1 in the Supplement). To reduce duplication of patients for whom ICD-10 codes may have been recorded multiple times (e.g., across healthcare encounters during treatment or upon relapse or recurrence), only the first documented ICD-10 code for each patient within the study period was used. We excluded codes specific to fungal and mycobacterial COM, as the diagnosis and treatment of these infections differ significantly from most common causes of bacterial COM. ICD-10 codes were used, whenever possible, to further classify cases based on COM anatomic sites, including upper-extremity, lower-extremity, vertebral column, and other (skull, ribs, sternum, and unspecified site) sites.

### 2.3 Covariates

Additional covariates included from the TriNetX database were age, sex, race/ethnicity, marital status, hospital region, and the following chronic conditions (using ICD-10 codes as outlined in Table S2): diabetes, hypertension, cardiovascular disease, chronic liver disease, chronic kidney disease, chronic lung disease, cerebrovascular disease, and chronic neurological condition. All covariates were assessed at baseline, i.e., at the time of the COM diagnosis. Chronic conditions could be present at or any time prior to the time of the COM diagnosis.

### 2.4 Data analysis

Baseline categorical characteristics were summarized as frequencies and percentages. To estimate the overall incidence of COM in the multicenter TriNetX database, we calculated the incidence of overall COM, anatomic-site-specific COM, and hospital-region-specific COM per 1000 adult patients recorded in the database (COM cases/population at risk) for each year from 2016 through 2022. Age- and sex-specific incidence of overall COM and anatomic-site-specific COM were also computed. We calculated the 95 % confidence intervals (CIs) for all estimates. Additionally, treating the year of diagnosis as an ordinal variable, we used the Cochran–Armitage test for trend to investigate potential trends in overall and stratum-specific COM incidence across the study period. We used a prespecified p -value threshold of 0.05 for statistical testing. This analysis was done using SAS version 9.4 (SAS Institute Inc., 2013).

## 3 Results

We identified a total of 93 324 adult patients with COM documented in the TriNetX database between 1 January 2016 and 31 December 2022 from approximately 80 healthcare organizations. The majority of affected patients were male (64.16 %). Lower-extremity COM was the most common anatomic site (44.87 %). Demographic and clinical characteristics for patients with COM are shown for each year of the study in Table 1; overall age and sex distributions in the TriNetX database are shown in Table S3.

**Table 1:** Chronic osteomyelitis (COM) cohort demographics.

Variable	Level	Overall		2016		2017		2018		2019		2020		2021		2022	
		( N = 93 324)		( N = 10 334)		( N = 13 207)		( N = 14 770)		( N = 15 698)		( N = 13 716)		( N = 15 065)		( N = 10 534)	
		n	%	n	%	n	%	n	%	n	%	n	%	n	%	n	%
Age	18–29 years	4095	4.39	543	5.25	624	4.72	690	4.67	671	4.27	601	4.38	592	3.93	374	3.55
	30–39 years	7801	8.36	982	9.50	1096	8.30	1299	8.79	1239	7.89	1110	8.09	1250	8.30	825	7.83
	40–49 years	12 431	13.32	1536	14.86	1799	13.62	2011	13.62	2041	13.00	1737	12.66	1922	12.76	1385	13.15
	50–59 years	21 371	22.90	2513	24.32	3315	25.10	3409	23.08	3644	23.21	3061	22.32	3218	21.36	2211	20.99
	60–69 years	24 754	26.52	2604	25.20	3454	26.15	3895	26.37	4131	26.32	3742	27.28	4098	27.20	2830	26.87
	70–79 years	16 278	17.44	1601	15.49	2156	16.32	2486	16.83	2813	17.92	2419	17.64	2807	18.63	1996	18.95
	80 + years	6594	7.07	555	5.37	763	5.78	980	6.64	1159	7.38	1046	7.63	1178	7.82	913	8.67
Sex	Female	33 439	35.83	3830	37.06	4755	36.00	5217	35.32	5702	36.32	4807	35.05	5417	35.96	3711	35.23
	Male	59 881	64.16	6504	62.94	8452	64.00	9552	64.67	9996	63.68	8907	64.94	9648	64.04	6822	64.76
Race/	American Indian/Alaska Native	631	0.68	70	0.68	110	0.83	103	0.70	101	0.64	92	0.67	90	0.60	65	0.62
ethnicity	Asian	935	1.00	73	0.71	103	0.78	161	1.09	154	0.98	146	1.06	182	1.21	116	1.10
	Black/African American	16 413	17.59	1988	19.24	2407	18.23	2507	16.97	2600	16.56	2402	17.51	2761	18.33	1748	16.59
	Hispanic/Latino	2342	2.51	300	2.90	315	2.39	332	2.25	377	2.40	345	2.52	383	2.54	290	2.75
	Native Hawaiian/Pacific Islander	85	0.09	16	0.16	7	0.05	16	0.11	15	0.10	13	0.10	10	0.07	8	0.08
	White	62 383	66.85	6800	65.80	8821	66.79	10 029	67.90	10 419	66.37	9154	66.74	9918	65.83	7242	68.75
	Unknown	10 535	11.29	1087	10.52	1444	10.93	1622	10.98	2032	12.94	1564	11.40	1721	11.42	1065	10.11
Marital	Married	16 487	17.67	1942	18.79	2238	16.95	2590	17.54	2712	17.28	2396	17.47	2756	18.29	1853	17.59
status	Single	23 479	25.16	2768	26.79	3221	24.39	3608	24.43	3903	24.86	3449	25.15	3808	25.28	2722	25.84
	Unknown	53 358	57.18	5624	54.42	7748	58.67	8572	58.04	9083	57.86	7871	57.39	8501	56.43	5959	56.57
Region	Midwestern United States	13 113	14.05	1752	16.95	1850	14.01	1958	13.26	2174	13.85	1871	13.64	1963	13.03	1545	14.67
	Northeastern United States	22 738	24.36	2553	24.70	2909	22.03	3765	25.49	4026	25.65	3517	25.64	3629	24.09	2339	22.20
	Southern United States	38 122	40.85	4285	41.47	5272	39.92	5858	39.66	6145	39.15	5641	41.13	6539	43.41	4382	41.60
	Western United States	12 739	13.65	1592	15.41	1743	13.20	1936	13.11	1995	12.71	1804	13.15	2022	13.42	1647	15.64
	Other/outside United States	2132	2.28	136	1.32	229	1.73	239	1.62	515	3.28	328	2.39	448	2.97	237	2.25
	Unknown	4480	4.80	16	0.16	1204	9.12	1014	6.87	843	5.37	555	4.05	464	3.08	384	3.65
Anatomic	Lower extremity	41 875	44.87	4679	45.28	6144	46.52	6977	47.24	7095	45.20	6029	43.96	6360	42.22	4591	43.58
site	Upper extremity	4135	4.43	527	5.10	603	4.57	630	4.27	740	4.71	573	4.18	620	4.12	442	4.20
	Vertebral column	31 994	34.28	3417	33.07	4318	32.69	4934	33.41	5174	32.96	4867	35.48	5564	36.93	3720	35.31
	Other sites	15 320	16.42	1711	16.56	2142	16.22	2229	15.09	2689	17.13	2247	16.38	2521	16.73	1781	16.91
Chronic	Diabetes	47 608	51.01	4735	45.82	6638	50.26	7663	51.88	8199	52.23	7079	51.61	7775	51.61	5519	52.39
conditions	Hypertension	60 667	66.13	6320	61.16	8402	63.62	9583	64.88	10 373	66.08	9139	66.63	9849	65.38	7001	66.46
	Cardiovascular disease	50 012	54.52	4944	47.84	6593	49.92	7816	52.92	8494	54.11	7754	56.53	8363	55.51	6048	57.41
	Chronic liver disease	2784	3.03	423	4.09	423	3.20	427	2.89	421	2.68	410	3.00	404	2.68	276	2.62
	Chronic kidney disease	26 922	29.35	2744	26.55	3680	27.86	4269	28.90	4589	29.23	4121	30.05	4402	29.22	3117	29.59
	Chronic lung disease	10 648	11.61	1337	12.94	1420	10.75	1596	10.81	1708	10.88	1640	11.96	1726	11.46	1221	11.59
	Cerebrovascular disease	13 485	14.70	1064	10.30	1568	11.87	1954	13.23	2265	14.43	2199	16.03	2546	16.90	1889	17.93
	Chronic neurological condition	13 913	15.17	1493	14.45	1888	14.30	2127	14.40	2297	14.63	2149	15.67	2291	15.21	1668	15.83

### 3.1 COM incidence in the TriNetX database

**Figure 1:**
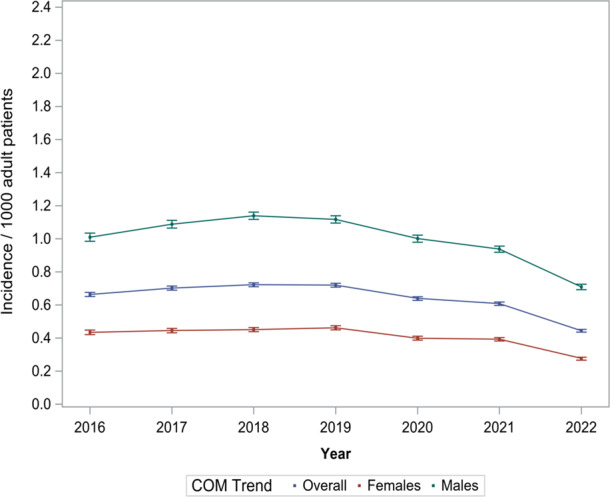
Overall and sex-specific incidence of chronic osteomyelitis (COM) per 1000 adult patients recorded in the multicenter TriNetX database (2016–2022).

**Figure 2:**
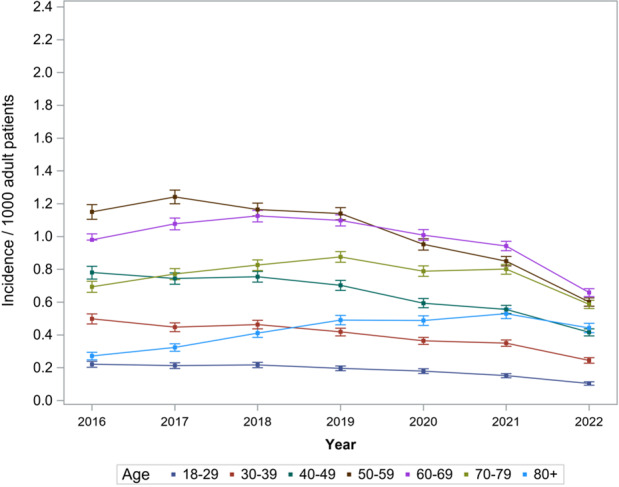
Age-specific COM incidence per 1000 adult patients recorded in the multicenter TriNetX database (2016–2022).

Overall, we observed a steady COM incidence rate over time (0.44–0.72 per 1000 patients recorded in the TriNetX database), with some indication of lower rates between 2020 and 2022 (Table S4 and Fig. 1). Males had a higher COM incidence compared to females (0.71–1.14 vs. 0.28–0.46 per 1000 patients). We also found steady COM trends across age groups. Patients aged 50 to 59 had a minor, non-significant reduction in the trend starting in 2019 ( p=0.62 ), while patients aged 70 or older appeared to have slightly increasing incidence over time (Table S4 and Fig. 2).

### 3.2 Region-specific COM incidence

COM incidence differed by region, and regional COM incidence varied over the study period. The Southern United States had the highest COM incidence (0.51–0.85 per 1000 adult patients), whereas the Midwestern United States had the lowest COM incidence (0.49–0.64 per 1000 adult patients) (Table 2). All regions experienced a reduction in incidence between 2019 and 2020.

**Table 2:** Region-specific and anatomic-site-specific COM incidence rates among all adult patients recorded in the TriNetX database (2016–2022).

Variable	Level	2016			2017			2018			2019			2020			2021			2022			$p^{c}$
																							(test for trend)
		Rate	95 % CI		Rate	95 % CI		Rate	95 % CI		Rate	95 % CI		Rate	95 % CI		Rate	95 % CI		Rate	95 % CI		
			LL	UL		LL	UL		LL	UL		LL	UL		LL	UL		LL	UL		LL	UL	
Region^a^	Midwestern United States	0.53	0.51	0.56	0.56	0.54	0.59	0.59	0.57	0.62	0.64	0.61	0.66	0.55	0.53	0.58	0.59	0.56	0.61	0.49	0.46	0.51	1
	Northeastern United States	0.63	0.61	0.66	0.65	0.62	0.67	0.69	0.67	0.72	0.71	0.69	0.73	0.64	0.62	0.66	0.55	0.53	0.57	0.36	0.35	0.38	0.8
	Southern United States	0.79	0.76	0.81	0.88	0.86	0.91	0.87	0.85	0.90	0.85	0.83	0.88	0.78	0.76	0.80	0.74	0.72	0.76	0.51	0.50	0.53	0.8
	Western United States	0.73	0.70	0.77	0.74	0.70	0.77	0.74	0.71	0.78	0.72	0.69	0.75	0.59	0.56	0.61	0.61	0.59	0.64	0.60	0.57	0.63	0.9
Anatomic site^b^	Lower extremity	0.30	0.29	0.31	0.33	0.32	0.34	0.34	0.33	0.35	0.33	0.32	0.33	0.28	0.27	0.29	0.26	0.25	0.26	0.19	0.19	0.20	0.9
	Upper extremity	0.03	0.03	0.04	0.03	0.03	0.04	0.03	0.03	0.03	0.03	0.032	0.04	0.03	0.03	0.03	0.03	0.02	0.03	0.02	0.02	0.02	1
	Vertebral column	0.22	0.21	0.23	0.23	0.22	0.24	0.24	0.23	0.25	0.24	0.23	0.24	0.23	0.22	0.23	0.22	0.22	0.23	0.16	0.15	0.16	0.9
	Other sites^d^	0.11	0.11	0.12	0.11	0.11	0.12	0.11	0.11	0.11	0.12	0.12	0.13	0.11	0.10	0.11	0.10	0.10	0.11	0.08	0.07	0.08	0.9

^a^ Incidence per 1000 adult patients recorded in the TriNetX database by region per year, shown alongside the lower limit (LL) and upper limit (UL) of the 95 % confidence interval (CI). ^b^ Incidence per 1000 adult patients recorded in the TriNetX database per year, shown alongside the LL and UL of the 95 % CI. ^c^ p values for the incidence trend of COM per 1000 adult patients recorded in the TriNetX database per year (2016–2022). ^d^ Other sites refer to the skull, ribs, sternum, and unspecified sites.

### 3.3 Anatomic-site-specific COM incidence

COM trends also differed by anatomic site. Lower-extremity COM had the highest incidence (0.19–0.34 per 1000 adult patients), followed by vertebral COM (0.16–0.24 per 1000 adult patients) (Table 2). While vertebral COM incidence remained stable during the initial onset of the COVID-19 pandemic (between 2019 and 2020), we observed a slightly decreasing trend of lower-extremity COM and upper-extremity COM incidence during that time (Table 2).

In general, males and patients aged 50–59 and 60–69 years had a higher incidence of all types of anatomical-site-specific COM (Tables S5 and S6), and males had a higher incidence of lower-extremity COM compared to other subtypes (Table S5). In female patients, vertebral COM and lower-extremity COM had comparable incidence, although vertebral COM appeared to become slightly more common than lower-extremity COM among females starting in 2020 (Table S5). Likewise, in patients aged 50–59 and 60–69 years, site-specific COM trends were generally steady over time, except for slight decreases observed starting in 2020 (Table S6). In contrast, patients aged 80 + had a slightly increasing trend in lower-extremity and vertebral COM between 2016 and 2022 (Table S6).

## 4 Discussion

Our evaluation of COM incidence trends in the TriNetX database between 2016 and 2022 revealed a slight decline in overall COM incidence starting in 2020. While there are no published reports that compare COM diagnosis trends before and during the COVID-19 pandemic, many existing studies assess the change in a major COM risk factor (i.e., trauma) during the pandemic (Sutherland et al., 2020; Patwary and Khattak, 2023; Yasin et al., 2021a; Hughes et al., 2023; Yasin et al., 2021b; Vandoros, 2022). Investigations from Tennessee, California, Florida, New York, and Massachusetts showed 15 %–49 % reductions in road traffic during the pandemic (2020) compared to a pre-pandemic period (Patwary and Khattak, 2023; Sutherland et al., 2020; Hughes et al., 2023). Meanwhile, a moderate negative correlation in vehicle collisions and vehicle injuries ( r∼-0.5 ) between 2019 and 2020 was observed in Florida, New York, and Massachusetts (Sutherland et al., 2020). Globally, reports from different countries, including China, the United Kingdom, Spain, the United Arab Emirates, and Greece, showed similar reductions in road traffic and trauma treatment during the pandemic (Yasin et al., 2021a, b; Vandoros, 2022). This reduction in vehicle collisions and crashes may be expected to reduce the overall risk for trauma-associated COM.

Similarly, competing events, such as death during the COVID-19 pandemic, might have affected the observed rates during this period, especially as COVID-19 mortality was strongly associated with diabetes, a major risk factor for COM (Lv et al., 2022; Yao et al., 2023), especially COM involving the lower extremities. An assessment of diabetes-related deaths between 2006 and 2021 found a 30 % increase in mortality during the pandemic (Lv et al., 2022). In another investigation examining death data between 2018 and 2022, similar findings were reported, with a 47.6 % increase in deaths observed where diabetes was one of multiple contributing causes (Yao et al., 2023).

Furthermore, the decline in overall COM incidence between 2019 and 2022 may be attributed to delays in COM diagnosis, which might have been caused or exacerbated by healthcare system overload. In a survey conducted in June 2020, 40.9 % of respondents reported delaying or avoiding healthcare for either emergency or routine care (Czeisler et al., 2020). In another assessment, a 10 % decline in United States emergency room visits for diabetes-related emergencies between March and May 2020 was reported (Lange et al., 2020). Globally, French and Canadian population-based analyses showed reductions in diabetic foot ulcer hospitalization, OM, and revascularization procedures during the pandemic compared to the pre-pandemic period (Mariet et al., 2021; de Mestral et al., 2022).

Our study's findings are generally consistent with prior research, including the demonstration of higher rates of COM among males compared to females and increasing rates of COM with age (Kremers et al., 2015; Walter et al., 2021). However, prior data showed a higher COM incidence among patients aged 80 years or older (Kremers et al., 2015), while our data demonstrated higher COM incidence rates among patients aged 50 to 69 years old for whom occupational or motor vehicle trauma and diabetes-related foot ulcers may be more common. This is supported by an analysis of the United States Diabetes Surveillance System Database (USDSSD), which showed an increasing trend of diabetes diagnosis between 2000 and 2022, with the highest reported trend among patients aged 65 or older (Nwachukwu et al., 2023).

We observed a higher incidence of COM in the southern region of the United States. To our knowledge, this study is the first to investigate regional COM trends in the United States, so it is unknown if this finding is consistent with previously examined OM data. However, regional distributions of COM-related risk factors strongly support this result (Nwachukwu et al., 2023). In the USDSSD study described above, the southern region had the highest reported diabetes diagnosis trend (Nwachukwu et al., 2023); this could explain the difference between our findings and the prior examination of OM in a county in the Midwestern United States (Kremers et al., 2015). Additionally, in an analysis of the National Hospital Ambulatory Medical Care Survey of nonfederal, general, and short-stay hospitals between 2017 and 2018, the southern region had the highest rates of motor-vehicle-accident-related emergency department visits (13.6 visits per 1000 persons) (Davis and Cairns, 2021), a difference which could contribute to trauma-related COM.

In our site-specific COM incidence assessment, lower-extremity COM had the highest incidence both overall and among males over time, while among females, vertebral and lower-extremity COM had comparable incidence with a possible shift toward vertebral COM beginning in 2020. Anatomic-site-specific COM incidence trends have not been previously established, although our findings align well with prior limited evidence. Our findings are consistent with a German investigation of lower-extremity OM burden between 2008 and 2018 (Walter et al., 2021). Different rates in lower-extremity trauma or diabetes may have contributed to the observed differences in site-specific COM incidence between males and females. Although we are unable to conclusively distinguish between subtypes of lower-extremity COM (e.g., long bone vs. foot), we expect this category to include a large number of cases of foot COM among patients with diabetes, and national diabetes statistics show a higher prevalence of diabetes among men compared to women (CDC, 2024). Meanwhile, an analysis of the Nationwide Inpatient Sample database showed a comparable incidence of vertebral OM admissions between males and females (Issa et al., 2018); this is in contrast to our findings, which demonstrated a higher incidence of vertebral COM among males than females. Furthermore, the stable incidence of vertebral COM among females throughout the pandemic might imply a minor increase in incidence, assuming that the pandemic had an equivalent impact on diagnosis rates of all COM subtypes in males and females.

There are some potential limitations to our study. First, COM incidence trends calculated from an electronic medical record database may not be representative of the general population. Incidence rates calculated within the TriNetX network, which is primarily composed of acute-care hospitals with multiple locations and large academic medical centers, may overestimate the incidence compared to other regional and community hospitals. This difference may be offset, at least in part, by the use of codes specific to COM in this study, which may underestimate COM in some cases (e.g., silent or undiagnosed COM or misclassification of COM as acute or unspecified OM). Furthermore, this study examines incidence trends within clinically and epidemiologically relevant strata (e.g., sex) over time. In the absence of another population-based OM dataset, our large, multicenter, and multi-region analysis provides an important steppingstone toward understanding national COM trends; however, further research is needed.

Second, we acknowledge that ICD-10 codes are less reliable for identifying COM cases compared to direct chart review or prospective studies, where more precise microbiological or histopathological findings may be recorded (Panteli and Giannoudis, 2016), and provide a less detailed characterization of other diagnostic features (e.g., distinctions between anatomic sites, such as long-bone COM in the lower extremities compared to foot COM; direct correlation with previous fractures or hardware placements at the same anatomic site; or consistently available information about microbiological causes). Direct comparison of ICD-10 codes against detailed patient records or diagnostic reasoning for individual cases was not feasible because of the anonymized nature of the data source. However, an analysis of TennCare Medicaid program data previously demonstrated that ICD codes have reasonable reliability for identifying OM (Wiese et al., 2018). Furthermore, we cannot directly assess changes in ICD coding practices over time, although we restricted our analysis to 2016–2022 to circumvent the impact of the healthcare coding system transition from ICD-9 to ICD-10 prior to 2016. While some conditions may have similar classifications in ICD-9 and ICD-10, this is not the case for OM. For example, unlike ICD-9, ICD-10 includes a code for vertebral OM.

Finally, we note that we deliberately included chronic recurrent multifocal osteomyelitis (CRMO), a rare autoimmune disease that mostly affects children and adolescents (Zhao et al., 2021) in our analysis. CRMO is exceptionally rare in adults (Yılmaz and İncesoy, 2024) and is a diagnosis of exclusion that requires ruling out an infectious cause, which is clinically difficult to achieve because pathogen identification is often difficult, even in bacterial COM (García Del Pozo et al., 2018a; Lew and Waldvogel, 2004). Thus, in ICD-10 coding data for adult patients, CRMO cannot be reliably distinguished from bacterial COM. Although CRMO accounts for about 4 % of all COM cases included in our incidence calculations, this decision could lead to minor overestimations in some rates. Despite these weaknesses, this study addresses a crucial question about COM and fills a significant knowledge gap about COM epidemiology and clinical care needs in the United States.

## 5 Conclusions

COM incidence in the TriNetX database was generally steady over the 2016–2022 period, with a possible slight reduction during the COVID-19 pandemic (after 2019). This study offers a unique snapshot of how the pandemic may have affected COM trends in the United States, particularly in terms of underlying risk factors, delays in diagnosis, and competing events. In addition, our results demonstrate the importance of vertebral COM, along with lower-extremity COM, in health systems and orthopedic infectious disease care in the United States.

## Supplement

10.5194/jbji-10-377-2025-supplement The supplement related to this article is available online at https://doi.org/10.5194/jbji-10-377-2025-supplement.

## Data Availability

Data are available upon reasonable request to the corresponding author and if/as approved by the institutional review board.

## Code availability

Code is available upon reasonable request to the corresponding author.

## Appendix

The supplement related to this article is available online at https://doi.org/10.5194/jbji-10-377-2025-supplement.
