# Supplementary material for: Incidence of chronic osteomyelitis between 2016 and 2022 in a large, multicenter database in the United States
Source: J Bone Jt Infect. 2025 Oct 15;10(5):377–84. doi: 10.5194/jbji-10-377-2025 (PMC12598500; doi:10.5194/jbji-10-377-2025)
Supplement: The supplement related to this article is available online at https://doi.org/10.5194/jbji-10-377-2025-supplement. [file jbji-10-377-2025-supplement.pdf]

Supplement of J. Bone Joint Infect., 10, 377–384, 2025
https://doi.org/10.5194/jbji-10-377-2025-supplement
© Author(s) 2025. CC BY 4.0 License.

Supplement of

Incidence of chronic osteomyelitis between 2016 and 2022 in a large, multicenter database in the United States

Rawabi Aljadani et al.

Correspondence to: Martha L. Carvour (martha-carvour@uiowa.edu)

The copyright of individual parts of the supplement might differ from the article licence.

Table S1. ICD-10 codes for chronic osteomyelitis, including site-specific codes where available

ICD-10 Code	Osteomyelitis Location
M46.2	Osteomyelitis of vertebra
M46.20	Osteomyelitis of vertebra, site unspecified
M46.21	Osteomyelitis of vertebra, occipito-atlanto-axial region
M46.22	Osteomyelitis of vertebra, cervical region
M46.23	Osteomyelitis of vertebra, cervicothoracic region
M46.24	Osteomyelitis of vertebra, thoracic region
M46.25	Osteomyelitis of vertebra, thoracolumbar region
M46.26	Osteomyelitis of vertebra, lumbar region
M46.27	Osteomyelitis of vertebra, lumbosacral region
M46.28	Osteomyelitis of vertebra, sacral and sacrococcygeal region
M86	Osteomyelitis
M86.3	Chronic multifocal osteomyelitis
M86.30	Chronic multifocal osteomyelitis, unspecified site
M86.31	Chronic multifocal osteomyelitis, shoulder
M86.311	Chronic multifocal osteomyelitis, right shoulder
M86.312	Chronic multifocal osteomyelitis, left shoulder
M86.319	Chronic multifocal osteomyelitis, unspecified shoulder
M86.32	Chronic multifocal osteomyelitis, humerus
M86.321	Chronic multifocal osteomyelitis, right humerus
M86.322	Chronic multifocal osteomyelitis, left humerus
M86.329	Chronic multifocal osteomyelitis, unspecified humerus
M86.33	Chronic multifocal osteomyelitis, radius and ulna
M86.331	Chronic multifocal osteomyelitis, right radius and ulna
M86.332	Chronic multifocal osteomyelitis, left radius and ulna
M86.339	Chronic multifocal osteomyelitis, unspecified radius and ulna
M86.34	Chronic multifocal osteomyelitis, hand
M86.341	Chronic multifocal osteomyelitis, right hand
M86.342	Chronic multifocal osteomyelitis, left hand
M86.349	Chronic multifocal osteomyelitis, unspecified hand
M86.35	Chronic multifocal osteomyelitis, femur
M86.351	Chronic multifocal osteomyelitis, right femur
M86.352	Chronic multifocal osteomyelitis, left femur
M86.359	Chronic multifocal osteomyelitis, unspecified femur
M86.36	Chronic multifocal osteomyelitis, tibia and fibula
M86.361	Chronic multifocal osteomyelitis, right tibia and fibula
M86.362	Chronic multifocal osteomyelitis, left tibia and fibula
M86.369	Chronic multifocal osteomyelitis, unspecified tibia and fibula
M86.37	Chronic multifocal osteomyelitis, ankle and foot
M86.371	Chronic multifocal osteomyelitis, right ankle and foot
M86.372	Chronic multifocal osteomyelitis, left ankle and foot
M86.379	Chronic multifocal osteomyelitis, unspecified ankle and foot
M86.38	Chronic multifocal osteomyelitis, other site
M86.39	Chronic multifocal osteomyelitis, multiple sites
M86.4	Chronic osteomyelitis with draining sinus
M86.40	Chronic osteomyelitis with draining sinus, unspecified site
M86.41	Chronic osteomyelitis with draining sinus, shoulder
M86.411	Chronic osteomyelitis with draining sinus, right shoulder
M86.412	Chronic osteomyelitis with draining sinus, left shoulder
M86.419	Chronic osteomyelitis with draining sinus, unspecified shoulder

M86.42	Chronic osteomyelitis with draining sinus, humerus
M86.421	Chronic osteomyelitis with draining sinus, right humerus
M86.422	Chronic osteomyelitis with draining sinus, left humerus
M86.429	Chronic osteomyelitis with draining sinus, unspecified humerus
M86.43	Chronic osteomyelitis with draining sinus, radius and ulna
M86.431	Chronic osteomyelitis with draining sinus, right radius and ulna
M86.432	Chronic osteomyelitis with draining sinus, left radius and ulna
M86.439	Chronic osteomyelitis with draining sinus, unspecified radius and ulna
M86.44	Chronic osteomyelitis with draining sinus, hand
M86.441	Chronic osteomyelitis with draining sinus, right hand
M86.442	Chronic osteomyelitis with draining sinus, left hand
M86.449	Chronic osteomyelitis with draining sinus, unspecified hand
M86.45	Chronic osteomyelitis with draining sinus, femur
M86.451	Chronic osteomyelitis with draining sinus, right femur
M86.452	Chronic osteomyelitis with draining sinus, left femur
M86.459	Chronic osteomyelitis with draining sinus, unspecified femur
M86.46	Chronic osteomyelitis with draining sinus, tibia and fibula
M86.461	Chronic osteomyelitis with draining sinus, right tibia and fibula
M86.462	Chronic osteomyelitis with draining sinus, left tibia and fibula
M86.469	Chronic osteomyelitis with draining sinus, unspecified tibia and fibula
M86.47	Chronic osteomyelitis with draining sinus, ankle and foot
M86.471	Chronic osteomyelitis with draining sinus, right ankle and foot
M86.472	Chronic osteomyelitis with draining sinus, left ankle and foot
M86.479	Chronic osteomyelitis with draining sinus, unspecified ankle and foot
M86.48	Chronic osteomyelitis with draining sinus, other site
M86.49	Chronic osteomyelitis with draining sinus, multiple sites
M86.5	Other chronic hematogenous osteomyelitis
M86.50	Other chronic hematogenous osteomyelitis, unspecified site
M86.51	Other chronic hematogenous osteomyelitis, shoulder
M86.511	Other chronic hematogenous osteomyelitis, right shoulder
M86.512	Other chronic hematogenous osteomyelitis, left shoulder
M86.519	Other chronic hematogenous osteomyelitis, unspecified shoulder
M86.52	Other chronic hematogenous osteomyelitis, humerus
M86.521	Other chronic hematogenous osteomyelitis, right humerus
M86.522	Other chronic hematogenous osteomyelitis, left humerus
M86.529	Other chronic hematogenous osteomyelitis, unspecified humerus
M86.53	Other chronic hematogenous osteomyelitis, radius and ulna
M86.531	Other chronic hematogenous osteomyelitis, right radius and ulna
M86.532	Other chronic hematogenous osteomyelitis, left radius and ulna
M86.539	Other chronic hematogenous osteomyelitis, unspecified radius and ulna
M86.54	Other chronic hematogenous osteomyelitis, hand
M86.541	Other chronic hematogenous osteomyelitis, right hand
M86.542	Other chronic hematogenous osteomyelitis, left hand
M86.549	Other chronic hematogenous osteomyelitis, unspecified hand
M86.55	Other chronic hematogenous osteomyelitis, femur
M86.551	Other chronic hematogenous osteomyelitis, right femur
M86.552	Other chronic hematogenous osteomyelitis, left femur
M86.559	Other chronic hematogenous osteomyelitis, unspecified femur
M86.56	Other chronic hematogenous osteomyelitis, tibia and fibula
M86.561	Other chronic hematogenous osteomyelitis, right tibia and fibula

M86.562	Other chronic hematogenous osteomyelitis, left tibia and fibula
M86.569	Other chronic hematogenous osteomyelitis, unspecified tibia and fibula
M86.57	Other chronic hematogenous osteomyelitis, ankle and foot
M86.571	Other chronic hematogenous osteomyelitis, right ankle and foot
M86.572	Other chronic hematogenous osteomyelitis, left ankle and foot
M86.579	Other chronic hematogenous osteomyelitis, unspecified ankle and foot
M86.58	Other chronic hematogenous osteomyelitis, other site
M86.59	Other chronic hematogenous osteomyelitis, multiple sites
M86.6	Other chronic osteomyelitis
M86.60	Other chronic osteomyelitis, unspecified site
M86.61	Other chronic osteomyelitis, shoulder
M86.611	Other chronic osteomyelitis, right shoulder
M86.612	Other chronic osteomyelitis, left shoulder
M86.619	Other chronic osteomyelitis, unspecified shoulder
M86.62	Other chronic osteomyelitis, humerus
M86.621	Other chronic osteomyelitis, right humerus
M86.622	Other chronic osteomyelitis, left humerus
M86.629	Other chronic osteomyelitis, unspecified humerus
M86.63	Other chronic osteomyelitis, radius and ulna
M86.631	Other chronic osteomyelitis, right radius and ulna
M86.632	Other chronic osteomyelitis, left radius and ulna
M86.639	Other chronic osteomyelitis, unspecified radius and ulna
M86.64	Other chronic osteomyelitis, hand
M86.641	Other chronic osteomyelitis, right hand
M86.642	Other chronic osteomyelitis, left hand
M86.649	Other chronic osteomyelitis, unspecified hand
M86.65	Other chronic osteomyelitis, thigh
M86.651	Other chronic osteomyelitis, right thigh
M86.652	Other chronic osteomyelitis, left thigh
M86.659	Other chronic osteomyelitis, unspecified thigh
M86.66	Other chronic osteomyelitis, tibia and fibula
M86.661	Other chronic osteomyelitis, right tibia and fibula
M86.662	Other chronic osteomyelitis, left tibia and fibula
M86.669	Other chronic osteomyelitis, unspecified tibia and fibula
M86.67	Other chronic osteomyelitis, ankle and foot
M86.671	Other chronic osteomyelitis, right ankle and foot
M86.672	Other chronic osteomyelitis, left ankle and foot
M86.679	Other chronic osteomyelitis, unspecified ankle and foot
M86.68	Other chronic osteomyelitis, other site
M86.69	Other chronic osteomyelitis, multiple sites

Table S2. ICD-10 codes for comorbidities

**Comorbidities**<br>(Present before or at the time of the chronic osteomyelitis diagnosis)	**ICD-10**
Diabetes	E08-E13
Hypertension	I10-I15, I10-I16
Cardiovascular disease	I25, I30-I52, I30-I5A
Chronic kidney disease	N18
Chronic liver disease	K70-K77
Chronic lung disease	J40-J47
Chronic neurological disease	G00-G99
Cerebrovascular disease	I60-I69

Table S3. TriNetX database demographics (2016-2022)

Variable	Level	Year 2016 (N=15,575,415)	2017 (N=18,808,608)	2018 (N=20,422,238)	2019 (N=21,799,357)	2020 (N=21,446,659)	2021 (N=24,758,867)	2022 (N=23,693,165)
**HCOs*, N**		76	78	79	80	80	82	81
**Age, n**	18-29	2,448,745	2,920,914	3,178,664	3,415,130	3,349,390	3,885,752	3,571,597
	30-39	1,969,314	2,446,259	2,800,424	2,955,984	3,043,667	3,564,530	3,360,604
	40-49	1,968,220	2,416,866	2,662,778	2,901,723	2,923,525	3,453,608	3,326,500
	50-59	2,185,018	2,669,533	2,927,186	3,195,967	3,213,750	3,784,067	3,675,172
	60-69	2,658,018	3,206,627	3,461,252	3,759,502	3,707,611	4,346,574	4,297,086
	70-79	2,306,652	2,794,671	3,009,381	3,209,568	3,065,460	3,502,930	3,401,791
	80+	2,039,450	2,353,738	2,382,551	2,361,486	2,143,257	2,221,406	2,060,415
**Sex, n**	Female	8,816,847	10,680,681	11,556,595	12,333,601	12,038,733	13,781,884	13,405,962
	Male	6,441,959	7,763,927	8,383,471	8,945,902	8,893,109	10,288,308	9,624,018
**Region, n**	Midwest	3,281,508	3,281,290	3,308,014	3,411,562	3,396,383	3,337,140	3,184,944
	Northeast	4,031,349	4,512,482	5,429,227	5,666,553	5,468,295	6,628,510	6,442,033
	South	5,443,446	5,960,537	6,710,174	7,197,905	7,266,021	8,860,510	8,553,715
	West	2,173,957	2,360,007	2,604,250	2,768,608	3,080,496	3,291,240	2,732,383

* HCOs: healthcare organizations

Table S4. Overall, age-, and sex-specific COM incidence among all adult patients recorded in the TriNetX database (2016-2022)

Variable	Level	2016			2017			2018			2019			2020			2021			2022			P** (Test for Trend)
			95% CI			95% CI			95% CI			95% CI			95% CI			95% CI			95% CI		
		Incidence*	Lower	Upper	Incidence*	Lower	Upper	Incidence*	Lower	Upper	Incidence*	Lower	Upper	Incidence*	Lower	Upper	Incidence*	Lower	Upper	Incidence*	Lower	Upper	
**Overall**		0.66	0.651	0.676	0.7	0.69	0.714	0.72	0.711	0.734	0.72	0.708	0.73	0.64	0.628	0.65	0.61	0.598	0.618	0.44	0.436	0.453	0.83
**Age**	18-29	0.22	0.203	0.24	0.21	0.196	0.23	0.22	0.201	0.233	0.2	0.182	0.211	0.18	0.165	0.194	0.15	0.14	0.164	0.1	0.094	0.115	0.82
	30-39	0.5	0.467	0.529	0.45	0.421	0.474	0.46	0.438	0.489	0.42	0.395	0.442	0.36	0.343	0.386	0.35	0.331	0.37	0.25	0.228	0.262	0.75
	40-49	0.78	0.741	0.819	0.74	0.71	0.778	0.76	0.722	0.788	0.7	0.672	0.733	0.59	0.566	0.622	0.56	0.531	0.581	0.42	0.394	0.438	0.71
	50-59	1.15	1.105	1.195	1.24	1.2	1.284	1.16	1.126	1.204	1.14	1.103	1.177	0.95	0.918	0.986	0.85	0.82	0.879	0.6	0.576	0.626	0.62
	60-69	0.98	0.979	1.017	1.08	1.041	1.113	1.13	1.09	1.16	1.1	1.065	1.132	1.01	0.977	1.042	0.94	0.914	0.971	0.66	0.634	0.682	0.8
	70-79	0.69	0.66	0.728	0.77	0.738	0.804	0.83	0.793	0.858	0.88	0.844	0.908	0.79	0.757	0.821	0.8	0.771	0.831	0.59	0.561	0.612	0.95
	80+	0.27	0.249	0.29	0.32	0.301	0.347	0.41	0.385	0.437	0.49	0.462	0.519	0.49	0.458	0.517	0.53	0.5	0.56	0.44	0.414	0.471	0.77
**Sex**	Female	0.43	0.421	0.448	0.45	0.432	0.458	0.45	0.439	0.464	0.46	0.45	0.474	0.4	0.388	0.411	0.39	0.382	0.403	0.28	0.267	0.285	0.85
	Male	1.01	0.985	1.034	1.09	1.065	1.112	1.14	1.117	1.162	1.12	1.095	1.139	1	0.98	1.022	0.94	0.919	0.956	0.71	0.692	0.725	0.8

* Incidence / 1000 adult patients recorded in the TriNetX database per year

** P values for incidence trend of COM / 1000 adult patients recorded in the TriNetX database per year (2016-2022)

Table S5. Sex-specific and anatomic site-specific COM incidence (2016-2022)

		Lower extremity				Upper extremity				Vertebral column				Other sites			
				95% CI				95% CI				95% CI				95% CI	
**Sex**	**Year**	**n**	**Incidence***	**Lower**	**Upper**	**n**	**Incidence***	**Lower**	**Upper**	**n**	**Incidence***	**Lower**	**Upper**	**n**	**Incidence***	**Lower**	**Upper**
**Females**	2016	1536	0.17	0.166	0.18	221	0.025	0.022	0.028	1371	0.16	0.15	0.16	702	0.08	0.074	0.086
	2017	1870	0.18	0.167	0.18	222	0.02	0.018	0.024	1818	0.17	0.16	0.18	845	0.08	0.074	0.084
	2018	2119	0.18	0.176	0.19	230	0.02	0.017	0.022	1970	0.17	0.16	0.18	898	0.08	0.073	0.083
	2019	2265	0.18	0.176	0.19	296	0.024	0.021	0.027	2123	0.17	0.16	0.18	1018	0.08	0.077	0.088
	2020	1788	0.15	0.142	0.16	213	0.018	0.015	0.02	1934	0.16	0.15	0.17	872	0.07	0.068	0.077
	2021	1939	0.14	0.134	0.15	245	0.018	0.016	0.02	2287	0.17	0.16	0.17	946	0.07	0.064	0.073
	2022	1355	0.1	0.096	0.11	159	0.012	0.01	0.014	1536	0.11	0.11	0.12	661	0.05	0.046	0.053
**Males**	2016	3143	0.49	0.47	0.5	306	0.048	0.042	0.053	2046	0.32	0.3	0.33	1009	0.16	0.15	0.17
	2017	4274	0.55	0.53	0.57	381	0.05	0.044	0.054	2500	0.32	0.31	0.33	1297	0.17	0.16	0.18
	2018	4858	0.58	0.56	0.6	399	0.048	0.043	0.052	2964	0.35	0.34	0.37	1331	0.16	0.15	0.17
	2019	4830	0.54	0.52	0.56	444	0.05	0.045	0.054	3051	0.34	0.33	0.35	1671	0.19	0.18	0.2
	2020	4240	0.48	0.46	0.49	360	0.04	0.036	0.045	2933	0.33	0.32	0.34	1374	0.15	0.15	0.16
	2021	4421	0.43	0.42	0.44	375	0.036	0.033	0.04	3277	0.32	0.31	0.33	1575	0.15	0.15	0.16
	2022	3236	0.34	0.32	0.35	283	0.029	0.026	0.033	2183	0.23	0.22	0.24	1120	0.12	0.11	0.12

* Incidence / 1000 (females/ males) adult patients recorded in the TriNetX database per year

Table S6. Age-specific and anatomic site-specific COM incidence (2016-2022)

Site	Year	18-29				30-39				40-49				50-59				60-69				70-79				80+			
				95% CI				95% CI				95% CI				95% CI				95% CI				95% CI				95% CI	
Site	Year	n	Incidence*	Lower	Upper	n	Incidence*	Lower	Upper	n	Incidence*	Lower	Upper	n	Incidence*	Lower	Upper	n	Incidence*	Lower	Upper	n	Incidence*	Lower	Upper	n	Incidence*	Lower	Upper
Lower extremity	2016	194	0.08	0.068	0.09	395	0.2	0.181	0.22	722	0.37	0.34	0.39	1212	0.55	0.52	0.59	1200	0.45	0.43	0.48	711	0.31	0.29	0.33	245	0.12	0.11	0.14
	2017	205	0.07	0.061	0.08	439	0.18	0.163	0.2	850	0.35	0.33	0.38	1694	0.63	0.6	0.66	1663	0.52	0.49	0.54	941	0.34	0.32	0.36	352	0.15	0.13	0.17
	2018	225	0.07	0.062	0.08	518	0.19	0.169	0.2	964	0.36	0.34	0.38	1775	0.61	0.58	0.63	1958	0.57	0.54	0.59	1092	0.36	0.34	0.38	445	0.19	0.17	0.2
	2019	227	0.07	0.058	0.075	502	0.17	0.155	0.18	925	0.32	0.3	0.34	1794	0.56	0.54	0.59	1962	0.52	0.5	0.54	1169	0.36	0.34	0.39	516	0.22	0.2	0.24
	2020	191	0.06	0.049	0.065	411	0.14	0.122	0.15	765	0.26	0.24	0.28	1446	0.45	0.43	0.47	1765	0.48	0.45	0.5	1007	0.33	0.31	0.35	444	0.21	0.19	0.23
	2021	167	0.04	0.036	0.049	460	0.13	0.117	0.14	810	0.23	0.22	0.25	1457	0.39	0.37	0.4	1881	0.43	0.41	0.45	1083	0.31	0.29	0.33	502	0.23	0.21	0.25
	2022	151	0.04	0.036	0.049	314	0.09	0.083	0.1	627	0.19	0.17	0.2	1025	0.28	0.26	0.3	1293	0.3	0.28	0.32	818	0.24	0.22	0.26	363	0.18	0.16	0.19
Upper extremity	2016	50	0.02	0.015	0.026	60	0.03	0.023	0.038	82	0.04	0.033	0.051	115	0.05	0.043	0.062	120	0.05	0.037	0.053	78	0.03	0.026	0.041	22	0.01	0.006	0.02
	2017	62	0.02	0.016	0.027	72	0.03	0.023	0.036	97	0.04	0.032	0.048	139	0.05	0.043	0.061	131	0.04	0.034	0.048	77	0.03	0.021	0.034	25	0.01	0.007	0.02
	2018	57	0.02	0.013	0.023	101	0.04	0.029	0.043	90	0.03	0.027	0.041	137	0.05	0.039	0.055	156	0.05	0.04	0.052	67	0.02	0.017	0.028	22	0.01	0.005	0.013
	2019	64	0.02	0.014	0.023	84	0.03	0.022	0.034	105	0.04	0.029	0.043	182	0.06	0.049	0.065	170	0.05	0.04	0.052	100	0.03	0.025	0.037	35	0.01	0.01	0.02
	2020	49	0.01	0.011	0.019	80	0.03	0.021	0.032	83	0.03	0.022	0.034	126	0.04	0.032	0.046	139	0.04	0.03	0.044	70	0.02	0.017	0.028	26	0.01	0.008	0.017
	2021	53	0.01	0.01	0.017	80	0.02	0.018	0.027	78	0.02	0.018	0.028	139	0.04	0.031	0.043	149	0.03	0.029	0.04	86	0.03	0.019	0.03	35	0.02	0.011	0.021
	2022	28	0.01	0.005	0.011	56	0.02	0.012	0.021	63	0.02	0.014	0.024	114	0.03	0.025	0.037	116	0.03	0.02	0.032	52	0.02	0.011	0.019	13	0.01	0.003	0.01
Vertebral column	2016	155	0.06	0.053	0.073	349	0.18	0.159	0.196	499	0.25	0.23	0.28	802	0.37	0.34	0.39	872	0.33	0.31	0.35	559	0.24	0.22	0.26	181	0.09	0.076	0.1
	2017	211	0.07	0.062	0.082	387	0.16	0.142	0.174	584	0.24	0.22	0.26	978	0.37	0.34	0.39	1132	0.35	0.33	0.37	777	0.28	0.26	0.3	249	0.11	0.093	0.12
	2018	237	0.08	0.065	0.084	473	0.17	0.154	0.184	684	0.26	0.24	0.28	1015	0.35	0.33	0.37	1218	0.35	0.33	0.37	962	0.32	0.3	0.34	345	0.15	0.13	0.16
	2019	207	0.06	0.052	0.069	432	0.15	0.132	0.16	664	0.23	0.21	0.25	1055	0.33	0.31	0.35	1333	0.35	0.34	0.37	1060	0.33	0.31	0.35	423	0.18	0.162	0.2
	2020	200	0.06	0.051	0.068	419	0.14	0.124	0.151	600	0.21	0.19	0.22	1010	0.31	0.29	0.33	1280	0.35	0.33	0.36	963	0.31	0.29	0.33	395	0.18	0.166	0.2
	2021	220	0.06	0.049	0.064	462	0.13	0.118	0.141	703	0.2	0.19	0.22	1123	0.3	0.28	0.31	1436	0.33	0.31	0.35	1166	0.33	0.31	0.35	454	0.2	0.186	0.22
	2022	82	0.02	0.018	0.028	286	0.09	0.075	0.095	474	0.14	0.13	0.16	734	0.2	0.19	0.21	974	0.23	0.21	0.24	801	0.24	0.22	0.25	369	0.18	0.161	0.2
Other sites	2016	144	0.06	0.049	0.068	178	0.09	0.077	0.104	233	0.12	0.103	0.134	384	0.18	0.158	0.19	412	0.16	0.14	0.17	253	0.11	0.096	0.12	107	0.05	0.043	0.062
	2017	146	0.05	0.042	0.058	198	0.08	0.07	0.092	268	0.11	0.098	0.124	504	0.19	0.172	0.21	528	0.16	0.151	0.18	361	0.13	0.116	0.14	137	0.06	0.048	0.068
	2018	171	0.05	0.046	0.062	207	0.07	0.064	0.084	273	0.1	0.09	0.115	482	0.17	0.15	0.18	563	0.16	0.149	0.18	365	0.12	0.109	0.13	168	0.07	0.06	0.081
	2019	173	0.05	0.043	0.058	221	0.08	0.065	0.085	347	0.12	0.107	0.132	613	0.19	0.177	0.21	666	0.18	0.164	0.19	484	0.15	0.137	0.16	185	0.08	0.067	0.09
	2020	161	0.05	0.041	0.055	200	0.07	0.057	0.075	289	0.1	0.087	0.11	479	0.15	0.136	0.16	558	0.15	0.138	0.16	379	0.12	0.111	0.14	181	0.08	0.072	0.097
	2021	152	0.04	0.033	0.045	248	0.07	0.061	0.078	331	0.1	0.086	0.106	499	0.13	0.12	0.14	632	0.15	0.134	0.16	472	0.14	0.123	0.15	187	0.08	0.072	0.096
	2022	113	0.03	0.026	0.037	169	0.05	0.043	0.058	221	0.07	0.058	0.075	338	0.09	0.082	0.1	447	0.1	0.094	0.11	325	0.1	0.085	0.11	168	0.08	0.069	0.094

* Incidence / 1000 adult patients of a specific age group recorded in the TriNetX database per year
